# Using the fused graphical lasso to explore the motivational self-system after a multimedia self-regulated learning training: a brief research report

**DOI:** 10.3389/fpsyg.2025.1414563

**Published:** 2025-03-11

**Authors:** Sarah M. Wolff, Jonathan C. Hilpert, Matthew L. Bernacki, Jeffrey A. Greene, Christy Strong

**Affiliations:** ^1^Department of Educational Psychology, Leadership and Higher Education, University of Nevada, Las Vegas, Las Vegas, NV, United States; ^2^School of Education, University of North Carolina at Chapel Hill, Chapel Hill, NC, United States; ^3^Department of Education, Korea University, Seoul, Republic of Korea; ^4^Department of Life Sciences, University of Nevada, Las Vegas, Las Vegas, NV, United States

**Keywords:** Gaussian graphical modeling, fused graphical lasso, motivation, self-regulated learning, complexity

## Abstract

**Introduction:**

The purpose of this study is to explore the effects of a randomized control trial designed to test the effect of a brief intervention used to improve self-regulated learning (SRL) in gateway biology courses using joint estimation of graphical models.

**Methods:**

Students (*N* = 265; *n* = 136) from three sections of a hybrid-format introductory biology course were randomly assigned to participate in the multimedia science of learning to learn or a multimedia control condition. All participants completed a self-report battery of motivational measures. Course performance data was also collected.

**Results:**

Network structures of motivation variables were estimated in two sub-groups (Treatment and Control). These networks showed a high level of correspondence in the relative magnitudes of the edge weights, however there were non-trivial differences in the edge weights between groups that may be attributed to the treatment and differences in predictability. While these findings suggest meaningful differences in motivational structures, the relatively small sample size may limit the stability of the estimated network models. The SRL strategy based interventions may have positioned the students motivationally to approach the challenging exam through activating the role of value and self-efficacy in their learning.

**Discussion:**

Many of the ways analyses of typical intervention studies are conducted ignore the underlying complexity of what motivates individuals. This study provides preliminary evidence how Gaussian Graphical Modeling may be valuable in preserving the integrity of complex systems and examining relevant shifts in variations between motivational systems between groups and individuals.

## Introduction

Improving students’ ability to self-regulate their own learning is important. Self-regulated learning (SRL) is a multifaceted process in which students actively engage in their learning, employing adaptive skills to achieve their goals through planning, performing, and reflecting on learning tasks (Pintrich, 2000; Schunk and Greene, 2017). Theories of SRL span various paradigms of cognition, affect, and behavior, with models differing in their conceptualization of metacognition, motivation, and emotion, as well as the structure and context of the learning process (Panadero, 2017). It is widely accepted, however, that the ability to monitor cognition, discriminate between well-learned and less well-learned knowledge, and implement strategies toward learning goals has significant implications for education across various aspects of learners’ behaviors, emotions, and cognitions (Sitzmann and Ely, 2011). Recognized as dynamic and interlinked components, SRL involves monitoring, controlling, and regulating cognition, motivation, volition, effort, and the self-system, all of which contribute to effective learning (Ben-Eliyahu and Bernacki, 2015; Winne and Hadwin, 1998; Zimmerman and Schunk, 2001).

Instructors are increasingly integrating active learning designs into their courses (Eddy and Hogan, 2014; Theobald et al., 2020), requiring students to acquire, rehearse, and evaluate knowledge through diverse activities, including reading, assignments, videos, and collaboration (Lombardi et al., 2021). This may pose increased challenges for learners (Azevedo et al., 2019; Bernacki, 2023). Many early undergraduate science learners report feeling underprepared for self-regulated learning (SRL) in such environments (Perez et al., 2014), often due to lack of familiarity, confidence, time, or preparation (Shekhar et al., 2020). In response, researchers have called for scaffolding methods to develop cognitive strategies and SRL practices (i.e., workshops, trainings, and embedded classroom activities) (Dignath et al., 2008) and theories on how to train such learners are emerging (Hattie and Donoghue, 2016; McDaniel and Einstein, 2020). Autonomous engagement is particularly important in student success online learning environments (Broadbent and Poon, 2015). Digital skill training programs are increasingly being explored as effective tools for supporting and enhancing SRL abilities (Theobald, 2021).

One critical aspect of self-regulated learning is the dynamic relations that occur when multiple self-regulated learning processes co-occur in context (Ben-Eliyahu and Bernacki, 2015), including how students are motivationally poised to engage in strategic learning (Efklides, 2011). Students’ perceptions of their academic endeavors, including their expectations, values, and goals need to be meaningfully shaped by their motivational valence (i.e., the degree to which these perceptions are experienced as positive or negative) and properly aligned in order for students to be successful. For example, recent work has used latent profile analysis to demonstrate how motivational variables with differing positive and negative associations combine when students are academically successful (Perez et al., 2023; Perez et al., 2019). Students with motivational profiles such as high confidence a low perceptions of costs associated with learning are more likely to earn better grades and score higher on exams (Perez et al., 2023). Complex systems perspectives on the study of motivation have described shifts in combinations as self-organizing psychological systems (Kaplan et al., 2012; Marchand and Hilpert, 2024).

Self-organization is the process by which a new order or pattern in a system arises from local interactions among parts of the system (Koopmans, 2020). In the context of motivation, self-organization can occur when the role, strength and direction of relationships among variables shifts as a result of a perturbation to the system (i.e., an intervention) leading to a more adaptive psychological state (Hilpert and Marchand, 2018). For example, after receiving training in self-regulated study skills, a student may feel more confident about their ability to perform well on a final exam (i.e., a shift toward stronger and more positively experienced self-efficacy) which may co-occur with a change in their goals for the exam from avoiding failure to performing well (i.e., a shift from avoidance goals to performance goals), and experience more positive emotions regarding taking the exam. The emergence of these changes in the student motivational system more poise them an increased change of academic success.

Although complex changes to the motivational system have been modeled using techniques such as latent profile analysis, these analytic techniques do not capture changes in the specific relationships between constructs. Network approaches, such as Gaussian Graphical Modeling (Epskamp, 2020), have become popular in other fields of psychology to study changes in constructs that underlie psychopathology (Epskamp et al., 2018a,b). These approaches have been useful not only because they maintain fidelity to the nature of complex systems themselves, (i.e., networks are the underlying structure of a complex system, see Mitchell, 2009), but also because they can be used to unpack the more specific changes between variables that occur within and between people over time (Costantini et al., 2019). Given the need for more research in this space, here we explore the effects of a randomized control trial designed to test the effect of a brief intervention used to improve self-regulated learning in gateway biology courses. Our previous work documents the details of the development and previous findings related to the intervention (Bernacki et al., 2020; Bernacki et al., 2021; Bernacki, 2023). For the current report, we show the effect of treatment on student motivation using network analysis. Our research questions were as follows:

RQ1: Is there evidence of improved self-organization in the motivational systems for students who received the treatment compared to those who engaged in control activities?

RQ2: For students who received the treatment, were their shifts in the betweeness, closeness, and strength of relationships among variables that aligned with motivational theory?

## Methods

### Participants and procedures

Students from three sections of a hybrid-format introductory biology course were randomly assigned to participate in the multimedia science of learning to learn or a multimedia control condition. Each module had three parts. Participants were 265 consenting undergraduates (27.17% male, 72.83% female). The ethnic/racial background of the students was as follows: 19.62% Asian/Asian American, 10.57% Black/African American, 35.85% Hispanic (Non-White), 12.83% Multiracial, 1.89% Pacific Islander, and 19.24% White/Caucasian. Of these students, 70 successfully completed all three parts of the intervention and 66 successfully completed all three parts of the control. Of these data, 17 participants had data missing in the treatment group and 16 had data missing in the control group. Because of the sample size limitations, the missing data were imputed using the random forest method via the missForest package (Stekhoven, 2022), which predicts missing values iteratively by leveraging the relationships between the observed variables in the larger dataset. These imputed data were retained for the subsequent analyses and demographic information is provided in Table 1.

**Table 1:** Sample demographic information by group.

	Control	Treatment
*n*	66	70
Age, mean years	20.3	20.6
% Sex		
Male	22.7	30.0
Female	77.3	70.0
% Ethnicity/race		
Black/African American	10.6	7.14
Asian	16.7	17.1
Hispanic or Latino	37.9	38.6
Native Hawaiian/Pacific Islander	6.1	–
White (Non-Hispanic Origin)	16.7	22.9
Two or more races	12.1	14.3

### Measures

#### Multimedia science of learning to learn

The multimedia science of learning to learn training is a redesign of a brief digital skill training program designed to enhance cognitive, metacognitive, and behavioral and environmental regulation strategies (Bernacki et al., 2020) wherein textual content was replaced with digital videos, an adjustment aimed to enhance learning efficiency by allowing learners to save time through video viewing instead of reading (Koedinger et al., 2012). Consisting of three modules, the program included a total of 12 videos covering cognitive study strategies, self-regulated learning techniques, and goal achievement strategies in biology. Activities within the modules aimed to promote knowledge rehearsal and deeper understanding of the video content, offering a more engaging and effective learning experience compared to static materials. Further details on both this program and the control alternative are provided in Bernacki (2023).

#### Achievement goal questionnaire-revised

Achievement goals were measured across nine items designed to measure mastery approach orientation (e.g., My aim is to completely master the material presented in this course), performance approach orientation (e.g., I am striving to do well compared to other students) and performance avoidance orientation toward learning (e.g., My goal is to avoid performing poorly compared to others) (Elliot and Murayama, 2008). Items were measured on a 7-point scale ranging from 1-strongly disagree to 7-strongly agree. These items demonstrate adequate subscale reliability, *α* = 0.84–0.94 (Elliot and Murayama, 2008).

#### Self-efficacy

Self-efficacy was measured with five items (e.g., I can do almost all the work in this course if I do not give up) taken from the Patterns of Adaptive Learning Scales (PALS; Midgley et al., 2000). Items were measured on a 6-point scale ranging from 1-strongly disagree to 6-strongly agree. The items demonstrate reliability, *α* = 0.78 (Midgley et al., 2000).

#### Perceived cost and value

Perceived cost and value were measured with 16 items adapted from Perez et al. (2014). Value was broken down into three factors with four indicators each, attainment value (e.g.), intrinsic value (e.g.), and utility value (e.g.) (Eccles and Wigfield, 1995). Cost was broken down into three factors with four indicators each, opportunity cost (e.g.), effort cost (e.g.) and psychological cost (e.g.) (Battle and Wigfield, 2003; Eccles, 1983). Items were measured on a 6-point scale ranging from 1-strongly disagree to 6-strongly agree. Subscales demonstrate adequate reliability, *α* = 0.75–0.93.

#### Metacognitive self-regulation

Metacognitive self-regulation was measured with 12 items from a subscale of the Motivated Strategies for Learning Questionnaire (MSLQ; Pintrich et al., 1991) designed to measure the planning, monitoring, and regulating of self-regulatory activities (e.g.). Items were measured on a 6-point scale ranging from 1-not at all true of me to 6-very true of me and demonstrate adequate reliability, *α* = 0.70 (Pintrich et al., 1991).

### Data analyses

Recent advancements in network analysis have addressed the challenge of estimating and comparing networks across different groups while preserving their unique characteristics (Costantini et al., 2019; Danaher et al., 2014; Guo et al., 2011). Traditional methods, such as estimating separate networks or using information criteria, fail to effectively leverage similarities between groups without obscuring their differences. Here, joint estimation of graphical models was conducted using the Fused Graphical Lasso (FGL). Building upon the graphical lasso methodology, the FGL introduces additional tuning parameters to penalize differences between group networks, facilitating the identification of shared edges while preserving group distinctions. This approach builds on traditional methods using partial correlation networks for cross-sectional data (Costantini et al., 2015), which rely on regularization techniques like the least absolute shrinkage and selection operator (lasso; Tibshirani, 1996) to handle overfitting and instability in estimating partial correlation matrices (Friedman et al., 2008). The FGL promotes network parsimony, enhances model fit by exploiting group similarities, and more accurately identifies true group differences (Danaher et al., 2014). The choice of tuning parameters in regularization is determined through methods like the Extended BIC (EBIC; Chen and Chen, 2008) or cross-validation (Krämer et al., 2009).

To explore stable individual differences and similarities between subjects in the treatment and control groups, two between-subject partial correlation networks were estimated using the FGL joint estimation technique in the R package *EstimateGroupNetwork* (Costantini et al., 2019). Between subject networks provide information on the underlying structure of differences between subjects and can be helpful for illuminating complex interactions between psychological variables within a system (Epskamp et al., 2018a,b). Note that while the FGL improves model fit by exploiting similarities, if true networks are substantially different, it behaves akin to estimating networks independently, enabling the emergence of true differences. The *qgraph* package (Epskamp et al., 2012) was used for network visualization and centrality estimates. Tuning parameter selection was conducted via EBIC and consistent with package *qgraph*’s function *EBICglasso*. Network structures were analyzed using the means of survey responses taken at the end of the semester for each individual.

The predictability of individual variables constituting the motivational system was assessed as the extent to which the variance of each variable is accounted for by the other nodes in the network (*R*^2^) using Mixed Graphical Models (MGM), implemented in R with the *mgm* package (Haslbeck and Waldorp, 2020). These predictability parameters were integrated into the FGL networks. Spearman correlation coefficients were computed between the lower triangular portions of the adjacency matrices of each network (edge weights) as a measure of overall similarity for each pair of networks. It quantifies the degree to which the rankings of the edge weights in one network correspond to the rankings in the other network. Additionally, mean connectivity values for each network were calculated and compared.

Centrality indices were computed for each joint estimated network to assess the prominence of nodes: (1) strength, quantifying a node’s direct connections, (2) closeness, evaluating a node’s proximity to others indirectly, (3) betweenness, assessing a node’s role in mediating communication along average paths between other nodes (Costantini et al., 2015; Opsahl et al., 2010), and (4) expected influence, representing the expected impact of each node on other nodes in the network. Nodes are connected to each other through edges, which represent associations between the entities they represent. The structure and properties of the network emerge from the arrangement and characteristics of these nodes and edges (Newman, 2010).

The accuracy of edge parameters and centrality estimates were assessed using the *bootnet* package (Epskamp et al., 2018a,b), employing a bootstrap sampling approach with 10,000-iterations. To gauge the stability of strength centrality metrics, we utilized the correlation stability (CS) coefficient. This involved iteratively correlating centrality metrics between the original dataset and subsamples containing progressively fewer participants. The CS coefficient indicates the maximum proportion of participants that can be removed while ensuring a 95% probability that the correlation between centrality metrics remains at least 0.7, ideally surpassing 0.5 (Epskamp et al., 2018a,b).

## Results

Data and code are available on Open Science Framework (OSF) at https://osf.io/f6qwc/?view_only=c35c8f70c9264c56949b139c206497ed (blinded for review).

### Preliminary analyses

A series of independent samples t-tests were run for the motivation variables collected at the start of the semester to establish baseline equivalence. Results are provided in Table 2. The t-tests suggest that there are no statistically significant differences between groups and effect sizes were negligible. Baseline network comparisons were too sparse for comparison.

**Table 2:** Means, standard deviations, and independent samples t-tests at baseline.

Variable	Control (*n* = 66)		Treatment (*n = 70*)		*t*(134)	*p*	Cohen’s *d*
	*M*	*SD*	*M*	*SD*			
Mastery approach	6.22	1.34	6.38	0.74	−0.82	0.41	0.14
Performance approach	5.71	1.26	5.78	1.01	−0.35	0.73	0.06
Performance avoidance	5.67	1.59	5.68	1.46	−0.04	0.97	0.01
Self-efficacy	4.90	0.82	5.06	0.61	−1.23	0.22	0.21
Opportunity cost	2.04	1.07	1.86	0.96	1.02	0.31	−0.18
Effort cost	2.15	0.97	2.09	0.98	0.37	0.71	−0.06
Psychological cost	3.53	1.28	3.68	1.13	−0.68	0.50	0.12
Attainment value	5.25	0.65	5.32	0.57	−0.68	0.50	0.12
Intrinsic value	4.68	0.67	4.84	0.54	−1.57	0.12	0.27
Utility value	4.94	1.00	5.01	0.82	−0.48	0.63	0.08
Metacognitive self-regulation	4.76	0.67	4.77	0.72	−0.09	0.93	0.02

Means, standard deviations and zero-order correlation matrices of the 11 motivation variables at the end of the semester and final exam are presented in Tables 3, 4 for the control and treatment groups, respectively.

**Table 3:** Means, standard deviations, and zero-order correlations for the control group (*n* = 66).

	1	2	3	4	5	6	7	8	9	10	11	12
1. Final exam	–											
2. Mastery approach	0.09	–										
3. Performance approach	0.12	0.65**	–									
4. Performance avoidance	0.08	0.46**	0.69**	–								
5. Self-efficacy	0.09	0.48**	0.46**	0.24	–							
6. Opportunity cost	−0.28*	−0.10	0.15	0.11	−0.12	–						
7. Effort cost	−0.07	−0.37**	0.01	0.04	−0.15	0.60**	–					
8 Psychological cost	−0.10	0.05	0.08	0.22	−0.16	0.57**	0.43**	–				
9. Attainment value	0.04	0.61**	0.37**	0.26	0.57**	−0.20	−0.32*	0.15	–			
10. Intrinsic value	−0.04	0.53**	0.32*	0.28	0.57**	−0.09	−0.28*	−0.10	0.60**	–		
11. Utility value	−0.19	0.52**	0.51**	0.31*	0.60**	0.16	−0.17	−0.06	0.59**	0.63**	–	
12. Metacognitive self-regulation	0.12	0.45**	0.32*	0.29*	0.39**	0.04	0.15	0.13	0.42**	0.38**	0.25	–
*M*	60.52	5.79	5.17	5.21	4.49	2.62	2.73	3.78	4.71	3.96	4.29	4.40
*SD*	27.72	0.97	1.24	1.52	0.85	1.31	1.12	1.14	0.82	1.05	1.13	0.69

*p < 0.05, **p < 0.01, ***p < 0.001.

**Table 4:** Means, standard deviations, and zero-order correlations for the treatment group (*n* = 70).

	1	2	3	4	5	6	7	8	9	10	11	12
1. Final exam	–											
2. Mastery approach	0.18	–										
3. Performance approach	0.21	0.69^**^	–									
4. Performance avoidance	0.24	0.63^**^	0.85^**^	–								
5. Self-efficacy	0.29^*^	0.57^**^	0.70^**^	0.50^**^	–							
6. Opportunity cost	−0.08	−0.52^**^	−0.43^**^	−0.43^**^	−0.36^**^	–						
7. Effort cost	−0.24	−0.35^**^	−0.29^*^	−0.21	−0.18	0.59^**^	–					
8 Psychological cost	0.19	0.19	0.22	0.26	0.02	0.37^**^	0.23	–				
9. Attainment value	0.24	0.68^**^	0.69^**^	0.60^**^	0.60^**^	−0.47^**^	−0.36^**^	0.17	–			
10. Intrinsic value	0.10	0.36^**^	0.23	0.16	0.42^**^	−0.38^**^	−0.10	−0.02	0.41^**^	–		
11. Utility value	0.15	0.32^*^	0.06	−0.07	0.41^**^	−0.14	−0.20	−0.00	0.45^**^	0.64^**^	–	
12. Metacognitive self-regulation	0.02	0.54^**^	0.45^**^	0.28^*^	0.40^**^	−0.31^*^	−0.28^*^	0.33^*^	0.33^*^	0.50^**^	0.31^*^	–
*M*	62.49	5.87	5.52	5.70	4.55	2.56	2.73	4.00	4.83	4.00	4.14	4.49
*SD*	26.22	0.92	1.11	1.10	0.91	1.23	0.99	1.11	0.81	1.12	1.31	0.87

*p < 0.05, **p < 0.01, ***p < 0.001.

Measurement work was conducted on all of the latent constructs. First, corrected item-total correlations were calculated for all items within their respective construct. Across the constructs, item-total correlations were consistently above 0.03 aside from metacognitive self-regulation which ranged from 0.17 to 0.73. Two of these items that were reverse scored produced corrected item-total correlations <0.03 and were dropped at this point. They also did not correlate with the other items. In addition, a scree test and parallel analysis indicated the presence of two factors rather than one. Inspection of the individual items revealed that items 2, 4, 5, and 6 related more closely to specific study habits and were reading related. Items 3, 7, 9, 10, 11, and 12 seemed to encompass a more global metacognition related to the course. Given the interest in metacognitive self-regulation over study specific habits, items 2, 4, 5, and 6 were dropped. The remaining items were well correlated.

Next, the items were subject to four separate confirmatory factor analyses models using a fixed-mean referent loading approach to identification where items were specified to load on factors in line with scale publishers’ hypotheses. For mastery learning, three factors were specified (performance avoidance, mastery orientation, and performance orientation), for expectancy value items, six factors were specified (opportunity cost, effort cost, psychological cost, attainment value, intrinsic value, and utility value), for metacognitive self-regulated learning one factor was specified, and for self-efficacy one factor was specified. The models all demonstrated acceptable fit to the data, Comparative Fit Index (CFI = 0.905–0.962), Tucker-Lewis Index (TLI = 0.890–0.943), and Standardized Root Mean Square Residual (SRMR = 0.040–0.069). Across models, the Root Mean Square Error of Approximation (RMSEA) values ranged from 0.075 to 0.254, however demonstrated wide 90% confidence intervals with lower bounds reaching 0.068. The confidence interval provides a range within which the true population RMSEA is likely to fall, with smaller and more narrow confidence intervals indicate greater precision in estimating the true RMSEA. With a smaller sample size, RMSEA is oversensitive in rejecting true population models (Hu and Bentler, 1999). Here, the wide confidence intervals indicate this may be affecting its precision (Brown, 2015). Standardized factor loadings were all statistically significant and moderate to large in size, ranging from 0.506 to 0.966. Taken together, and provided the theoretical permissibility of the parameter estimates and sample size considerations, the models provided plausible representations of the underlying structure of the five constructs of interest. Cronbach’s alpha ranged from 0.89 to 0.91, indicating good reliability. Means on all subscales were calculated and used for subsequent analyses.

RQ1: Is there evidence of improved self-organization in the motivational systems for students who received the treatment compared to those who engaged in control activities?

#### Network structure

**Figure 1:**
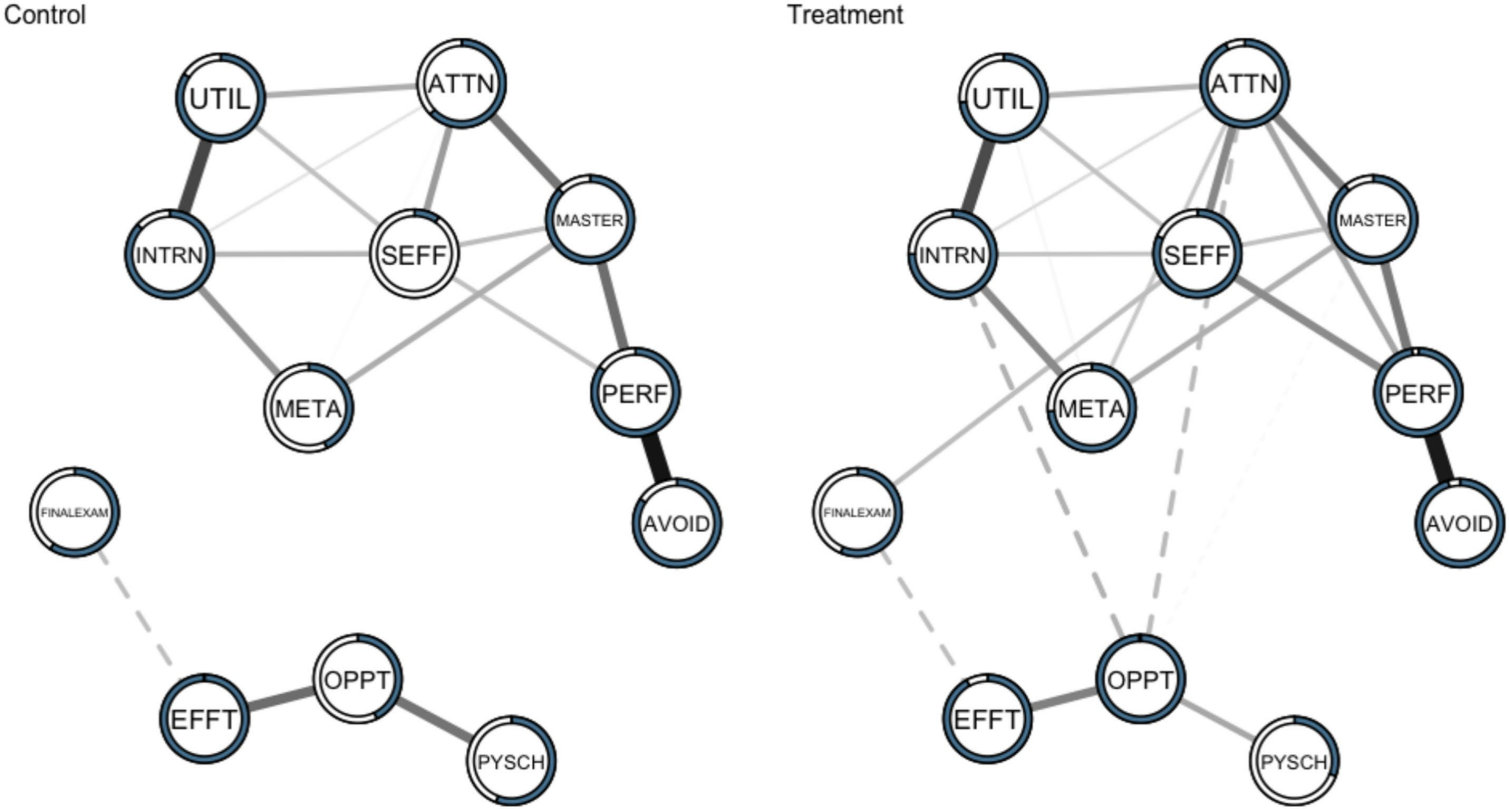
Networks of the motivation variables and final exam in the Control (left; *n* = 66) and Treatment (right; *n* = 70) samples. Dashed lines represent negative associations while solid lines indicate positive associations. Predictability (the proportion of variance in each variable that can be explained by all the other variables) is represented by the shaded area in the pie chart. UTIL, Utility Cost; SEFF, Self-Efficacy; PSYCH, Psychological Cost; PERF, Performance Approach Orientation; OPPT, Opportunity Cost; META, Metacognitive Self-Regulation; MASTER, Mastery Approach Orientation; INTRN, Intrinsic Value; EFFT, Effort Cost; AVOID, Performance Avoidance Orientation; ATTN, Attainment Value.

Network structures of motivation variables were estimated in two sub-groups: (1) students who participated and completed the three part multimedia intervention (Treatment) and (2) students who participated and completed the three part multimedia control activities (Control). A non-paranormal transformation was applied to the data to relax normality assumptions (Liu et al., 2009). The analyses were conducted on multiple sets of imputed data. It should be noted that the small sample size, combined with imputation and data transformation, introduces variability into our results. The networks presented here represent one possible solution. These networks are presented visually in Figure 1.

The motivational system descriptively explained a larger proportion of the variance of variables in the Treatment (mean explained variance 79.82%) versus Control participants (mean explained variance 66.27%). The overall similarity was assessed by computing the correlations between the edge weights across networks for each pair of networks (*r* = 0.88). This means that as the edge weights increase in one network, they tended to increase in the other network as well, and vice versa. The mean connectivity values were both 0.039. Based on these metrics, the networks in the control and treatment groups exhibit similarity.

While the networks may possess a high level of correspondence in the relative magnitudes of the edge weights between the two networks, there could still be differences in specific edges or connections between nodes. These differences might not be captured adequately by measures such as the mean connectivity or a correlation coefficient alone. To explore differences between the networks, we examine edge-wise comparisons and centrality measures.

In the Control sample, 18 of 66 possible edges (27.27%) were estimated to be above zero. This is notably different than the Treatment sample in which 24 of 66 possible edges (36.36%) were estimated to be above zero. It suggests a relatively larger number of edges play significant roles in connecting different nodes and controlling the flow of information or interactions within the network for the Treatment condition. These edges likely act as bridges or bottlenecks, influencing the overall network structure and dynamics. In the Control sample, absolute edge values ranged from −0.104 (effort cost with final exam) to 0.457 (performance orientation and performance avoidance). In the Treatment sample, absolute edge values ranged from −0.125 (perceptions of intrinsic value with opportunity cost) to 0.438 (performance approach orientation and performance avoidance orientation). All edges present in the Control network were also present in the Treatment network. There are also notable absences between nodes in the both groups. This suggests that some measured variables may have acted statistically independent when considering all other variables in the motivational system (their partial correlation is zero), or that there was not enough statistical power to detect a connection between them. In terms of unique edges in the Treatment condition, self-efficacy was connected to final exam (0.107), opportunity cost was marginally connected to mastery orientation (−0.009), attainment value was connected to performance approach orientation (0.150), opportunity cost was connected to attainment value (−0.112) and intrinsic value (−0.125), and metacognitive self-regulation were marginally connected to utility value (0.015).

RQ2: For students who received the treatment, where their shifts in the betweeness, closeness, and strength of relationships among variables that aligned with motivational theory?

#### Centrality indices

**Figure 2:**
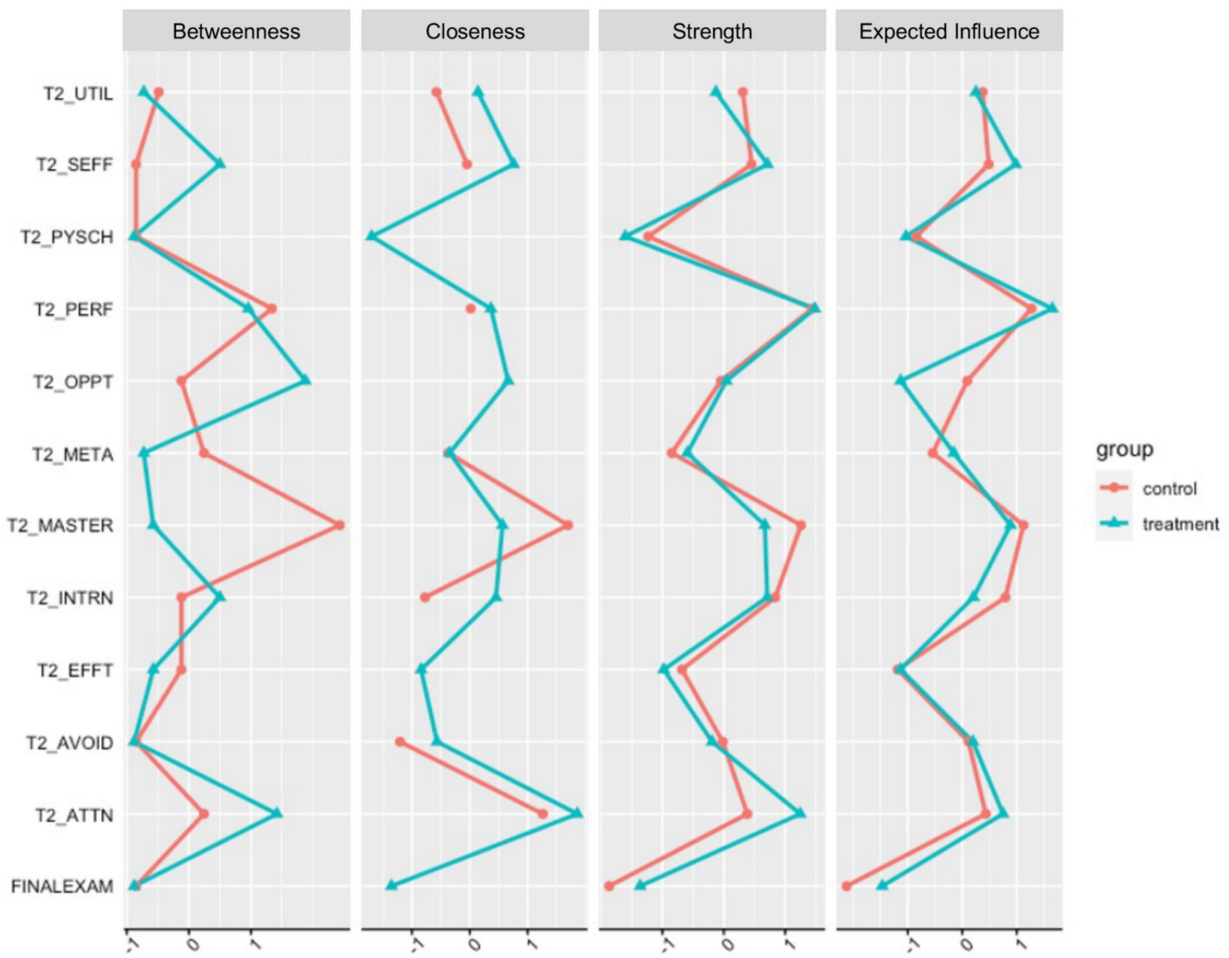
Nodes centralities for each of the variables in the motivational system for the Control (red; *n* = 66) and Treatment (blue; *n* = 70) samples. For ease of comparison, centrality values were standardized (z-scored) in each sample. UTIL, Utility Cost; SEFF, Self-Efficacy; PSYCH, Psychological Cost; PERF, Performance Approach Orientation; OPPT, Opportunity Cost; META, Metacognitive Self-Regulation; MASTER, Mastery Approach Orientation; INTRN, Intrinsic Value; EFFT, Effort Cost; AVOID, Performance Avoidance Orientation; ATTN, Attainment Value.

Without understanding the reliability of the network structure and the consistency of centrality estimates, it is difficult to determine if the variations in centrality estimates are meaningful or not. We calculated the maximum drop proportions needed to retain a correlation of 0.7 in at least 95% of the samples for various network metrics using bootstrap network estimation methods so that the spread of parameter and centrality estimates could be assessed. Simulation studies suggest that a correlation stability (CS) coefficient should ideally be above 0.5 and not below 0.25 to interpret centrality differences meaningfully (Epskamp et al., 2018a,b). CS coefficients suggest that the jointly estimated between-network edge centrality estimates (0.500) and strength centrality estimates (0.500) demonstrate adequate stability. CS coefficients for betweenness (0.051) and closeness (0.147), however, were below the recommended threshold for interpretability. The small sample size is likely to blame as networks with increasing sample sizes are estimated more accurately. In addition, sparsity in the network structure (when many edge-weights are expected to equal zero) can introduce bias in the bootstrapping (Epskamp et al., 2018a,b). We present all of these metrics here with this caution and emphasize that betweenness and closeness may not be interpretable with the present sample. Figure 2 shows centrality indices for all variables for both samples.

## Discussion

The purpose of this study was to explore the application of a novel network analysis technique (Fused Graphical Lasso; Costantini et al., 2019; Danaher et al., 2014) to examine the effects of a brief multimedia self-regulated learning intervention in gateway biology courses on the motivational self-system. We emphasize that the results presented are exploratory in nature and should not be interpreted with generalizability in mind. The small sample size presents a severe limitation to the reliability of the results. As noted by Danaher et al. (2014), complex network analysis techniques like Fused Graphical Lasso do not function well with small samples, leading to high false discovery rates. We offer an interpretation of the networks here to demonstrate the potential of using network analysis to uncover subtle differences in motivational dynamics between intervention and control groups, providing a more nuanced perspective on treatment effects that may not be evident through traditional statistical approaches.

Our findings suggest that, while the treatment and control networks remained correlated, there were non-trivial differences in the edge weights between groups that may be attributed to the treatment. In the control group, effort cost in the motivation system is related to final exam performance, and the three cost variables are disconnected from the rest of the motivational system. These findings suggest that for students who did not receive treatment, sunken effort was negatively associated with exam performance—i.e., effort that is expended without reward is costly (Inzlicht et al., 2018). However, we saw the emergence of a statistical relationship between self-efficacy and final exam performance in the treatment group that was not present in the control group, accompanied by an increase in the amount of variance explained in self-efficacy. The increasing role of self-efficacy in the treatment group co-occurred with the emergence of statistical relationships between attainment vale and performance orientation (+), attainment value and metacognition (+), attainment value and opportunity cost (−), as well as intrinsic motivation and opportunity cost (−). The emergence of these edges led to higher betweeness values for attainment value, opportunity cost, and self-efficacy in the treatment group. These changes may be indicative of a self-organizing motivational system, where the treatment simultaneously enhanced confidence, raised perceptions of attainment value, and lowered the perception that other opportunities were more important than preparing for the final exam. The SRL strategy based interventions may have positioned the students motivationally to approach the challenging exam through activating the role of value and self-efficacy in their learning.

While these results are exploratory, there are important potential implications for future research. Evidence of improved academic achievement after SRL interventions in online and digitally rich hybrid classes is mixed and the impact of these interventions on achievement produces a wide range of effect sizes, most typically hovering around moderate to small (Heikkinen et al., 2023; Theobald, 2021; Xu et al., 2023). In the current sample we did not find a significant difference between treatment and control on mean level course performance, likely due to insufficient power. In addition, there were no significant differences in motivational variables between the groups in t-tests, though sample size is likely a consideration. Even so, many of the ways analyses of typical intervention studies are conducted ignore the underlying complexity of what motivates individuals (Marchand and Hilpert, 2024). Here we see promising evidence to suggest that students who received the treatment were likely better poised motivationally to succeed on the final exam in the class. While this did not translate into significant exam differences overall, the FGL provided a way to look more closely at treatment effects that may otherwise be missed in the pursuit of a significance threshold.

These findings suggest that SRL interventions may be most effective when they simultaneously target multiple facets of motivation, such as strengthening self-efficacy while reducing perceived opportunity costs. Instructors and instructional designers may consider integrating SRL strategies that explicitly address these motivational components to better support students in challenging academic settings. Interventions may be made more effective by incorporating reflective exercises that help students recognize their progress and align their learning goals with personal values, thereby reinforcing motivation throughout the course. This study also provides preliminary evidence how Gaussian Graphical Modeling (Epskamp, 2020), may be valuable in preserving the integrity of complex systems and examining relevant shifts in variations between motivational systems between groups and individuals. Future research should aim for larger sample sizes to enhance the robustness of network analysis findings and to further validate the application of these methods in understanding self-regulated learning interventions.

## Data Availability

The datasets presented in this study can be found in online repositories. Data and code are available on Open Science Framework (OSF) at https://osf.io/f6qwc/?view_only=ca29cf78206d4034858056428c20e62f.
